# Cholecystectomy - a potential selection bias in studies assessing the metabolic effects of bariatric surgeries

**DOI:** 10.1038/s41598-020-66688-1

**Published:** 2020-06-30

**Authors:** Natasha Mendonça Machado, Camila de Siqueira Cardinelli, Tong Shen, Marco Aurélio Santo, Raquel Susana Torrinhas, Dan Linetzky Waitzberg

**Affiliations:** 1 Department of Gastroenterology, Laboratory of Nutrition and Metabolic Surgery of the Digestive Tract (LIM 35), Faculdade de Medicina FMUSP, Universidade de Sao Paulo, Sao Paulo, SP Brazil; 2 West Coast Metabolomics Center, University of California, Davis, CA United States; 3 Digestive Surgery Department. Hospital das Clinicas HCFMUSP, Faculdade de Medicina, Universidade de Sao Paulo, Sao Paulo, SP Brazil

**Keywords:** Metabolomics, Gastroenterology, Prognostic markers

## Abstract

Bile acids (BAs) are key mediators of the glycemic control after bariatric surgeries. Cholecystectomy modifies the kinetics of BAs, and whether this procedure influences the BAs pool and its metabolic response to bariatric surgeries is not known. We used targeted and untargeted metabolomics to assess whether cholecystectomy influenced plasma and fecal BAs fluctuations and the systemic metabolomic profile after Roux-en-Y gastric bypass (RYGB). Women with obesity and type 2 diabetes were included. Sample collections and clinical evaluations were performed before and 3 months after RYGB. RYGB influenced 9 fecal and 3 plasma BAs in patients with cholecystectomy (p ≤ 0.05). Comparisons between patients with and without cholecystectomy revealed different concentrations of 4 fecal and 5 plasma BAs (p ≤ 0.05). Cholecystectomy impacted the global metabolomics responses to RYGB, and patients who underwent the gallbladder removal also lacked some significant improvements in clinical markers, primarily the lipid profile. By affecting the BAs concentrations, cholecystectomy seems to alter the systemic metabolic response to RYGB. Therefore, cholecystectomy may act as a bias in assessments of the metabolic effects of bariatric surgeries and their relationships with clinical outcomes.

## Introduction

Bile acids (BAs) are key signaling molecules for glucose, fat and energy metabolism and act via the nuclear farnesoid X receptor (FXR) and the Takeda G protein receptor 5 (TGR5) in multiple organs^1^. The BAs pool may encompass crosstalk between the systemic environment and gut bacteria. Hepatocytes secret primary BAs, which are drained and concentrated in the gallbladder and properly released to the intestine for deconjugation and 7α-dehydroxylated by resident bacteria to form secondary BAs^2^.

The gallbladder is more than just a BA reservoir, and it is rich in the ileal hormone FGF19, which participates in the filling control of the gallbladder and the inhibition of BA synthesis^3^. Gallbladder removal via cholecystectomy may double the fasting enterohepatic recirculation rate of BAs and promote a potential higher exposure to cell surface and nuclear BA receptors^4^. These changes may influence lipid and carbohydrate metabolic pathways and are associated with an increased risk of diseases related to metabolic syndrome and insulin resistance^5^.

Due to the presence or risk of gallstone disease, cholecystectomy is generally performed in bariatric patients^6^. Changes in the BA pool were reported after bariatric surgery involving gastric bypass, and BAs are potential mediators of the metabolic effects of surgery, including early glycemic homeostasis^7,8^. Whether cholecystectomy influences the BAs pool of obese patients and its response to bariatric surgeries is not known, and studies on this topic generally do not consider the procedure as a criterion for patient selection.

As part of the SURmetaGIt study^9^, we evaluated the effects of Roux en-Y gastric bypass (RYGB) on plasma and fecal BAs fluctuations and its correlation with the remission of type 2 diabetes (T2D). A small group of our patients underwent cholecystectomy due to gallstone disease. During our initial analysis, we observed significant deviations in the dataset distribution of fecal and plasma BAs after RYGB, which disappeared when the data from patients with cholecystectomy were excluded. Therefore, we studied the BAs profile in different patient subgroups according to the presence or absence of cholecystectomy. This study reports our findings and shows that gallbladder removal impacted plasma and fecal BAs, systemic metabolomics and the clinical responses to RYGB. These findings suggest that cholecystectomy may be a source of bias in studies assessing the BAs profile and its metabolic effects after bariatric surgeries.

## Methods

### Patients and sample collection

Our study’s protocol followed the Helsinki Declaration and was approved by the local ethics committee (Comissão de Ética para Análise de Projetos de Pesquisa - CAPPesq 1011/09) and registered at www.ClinicalTrials.gov (NCT01251016). After signing written informed consent, women with obesity and T2D (aged 18–60 years) who were admitted for elective RYGB at the Gastrointestinal Surgery Division of the Hospital das Clínicas of University of Sao Paulo, School of Medicine (HC-FMUSP) were recruited. Patients met very restricted selection criteria and underwent a standardized RYGB, as described in the SURMetaGIT protocol^9^. We included patients without cholecystectomy and patients that underwent cholecystectomy prior or during RYGB, according to routine medical indications.

All patients were assessed for plasma BAs profiles, global metabolomics and clinical characteristics. A subset of patients was assessed for fecal BAs. All analyses were performed in samples collected before and 3 months after RYGB. Blood samples were collected after 12-hour fasting in EDTA-containing tubes. Plasma was obtained after centrifugation (10 min, 2,800 rpm, 4 °C) and kept frozen (−80 °C) until analysis. Fecal samples were collected in designated containers at the patients’ homes and immediately frozen at −20 °C. On the same day, samples were transported to our laboratory under controlled temperature, homogenized and aliquoted following the International Microbiome Standard IHMSSOP06 (http://www.microbiome-standards. org) and as described elsewhere^10^. Samples were stored at −80 °C until analysis.

### Bile acid profiles

Concentrations of cholic acid (CA), chenodeoxycholic acid (CDCA), deoxycholic acid (DCA), glycocholic acid (GCA), glycochenodeoxycholic acid (GCDCA), glycodeoxycholic acid (GDCA), glycolithocholic acid (GLCA), glycoursodeoxycholic acid (GUDCA), lithocholic acid (LCA), taurocholic acid (TCA), taurochenodeoxycholic acid (TCDCA), taurodeoxycholic acid (TDCA), taurolithocholic acid (TLCA), and tauroursodeoxycholic acid (TUDCA) were measured in both fecal and plasma samples by mass spectrometry-based targeted metabolomics.

### Targeted plasma bile acid profile

Plasma BAs profile was measured at the West Coast Metabolomics Center (metabolomics.ucdavis.edu) at the University of California, Davis, USA. Samples (50 µL) were treated with 5 µL anti-oxidants and 0.2 mg/mL butylated hydroxytoluene/EDTA in methanol:water (1:1, v/v), followed by spiking with 25 µL of a 1000 nM deuterium-labeled surrogate standard cocktail. Thereafter, 25 µL of 100 nM 12-[(cyclohexylcarbamoyl) aminododecanoic acid (CUDA) and 1-phenyl-3-hexadecanoic acid urea (PHAU) were added as external standards, followed by 125 μL acetonitrile/methanol (1:1, v/v) to precipitate proteins. Samples were centrifuged (15,000 rcf for 5 min at 4 °C), and the supernatants were filtered using a 96-well polyvinylidene difluoride (PVDF) plate. Deuterated surrogate standards included deoxycholic-2,2,4,4-d4 acid (GCA-d4), tauroursodeoxycholic acid (TCDCA-d4), cholic acid (CA-d6), glycochenodeoxycholic acid (GCDCA-d4), chenodeoxycholic acid (CDCA-d4), deoxycholic acid (DCA-d4) and lithocholic acid (LCA-d4). Five microliters of the extracts were injected.

BA concentrations were measured using a Waters ACQUITY liquid chromatography (LC) UPLC system (Waters Corporation, Milford, MA, USA) coupled to an AB Sciex 4000 Q-Trap triple quadrupole-linear ion trap hybrid mass spectrometer (MS). Chromatographic separation was attained in 18 min and was achieved using a Waters ACQUITY UPLC BEH C18 column (100 × 2.1 mm; 1.7 μm). The column was maintained at 45 °C with a flow rate of 0.4 mL/min. The mobile phases consisted of (A) water with 0.1% formic acid and (B) acetonitrile with 0.1% formic acid. Gradients were as follows: 0–0.5 min, 10% B; 0.5–1 min, 10–20% B; 1–1.5 min, 20–22.5% B; 1.5–4.3 min, 22.5–30% B; 4.3–4.6 min, 30–30.4% B; 4.6–11 min, 30.4–40% B; 11–12.5 min, 40–95% B; 12.5–14 min, 95% B; 14–14.5 min, 95-10% B; and 14.5–18 min, 10% B.

Multiple Reaction Monitoring (MRM) was performed in negative electrospray ionization mode. The collision-induced dissociation (CID) gas and curtain gas were N_2_. The ESI source parameters were optimized as follows: temperature, 600 °C; capillary voltage, −4500 V; Turbo ion source gas 1 and 2 (GS1, GS2), both at 30 psi; and curtain gas (CUR), 35 psi. The declustering potential (DP), entrance potential (EP), collision cell exit potential (CXP), dwell time, and collision energy (CE) were optimized for the optimum response for each analyte. The BAs were detected and quantified by generating seven-point intensity-to-concentration calibration curves. Samples were randomized for injection.

### Targeted fecal bile acid profile

Fecal BA metabolomic analysis was performed using a BA kit (Biocrates Life Sciences) according to the manufacturer’s instructions^11^. Briefly, sample preparation for metabolomics a mix of internal standards was pipetted into a 96-well filter plate. Samples were pipetted on the plate and dried with nitrogen, followed by the addition of 100 μL of methanol. Then, the combi-plate was shaken for 20 min and centrifuged to elute the methanol extract into the lower receiving deep-well plate. Sixty microliters of Milli-Q® water was added, and the plate was shaken vigorously. The fecal profile of BAs was acquired by liquid chromatography coupled to tandem mass spectrometry (LC-MS/MS) system, and multiple reaction monitoring (MRM) detection was performed in negative ESI mode. Samples were normalized according to the fecal sample weight (pmol/mg feces) and BA concentrations were determined in nM. Biocrates’ MetIDQTM software were used to data exportation.

### Untargeted plasma metabolomic analysis

To provide a comprehensive overview of the metabolic alterations induced by RYGB and affected by cholecystectomy, plasma samples were also analyzed using untargeted metabolomics. Metabolites were measured using a 6530 Accurate-Mass Q-TOF LC/MS and Agilent 1290 Infinity II LC System (Agilent Technologies), a combination of ultraperformance liquid chromatography (UPLC) with time of flight (TOF) tandem mass spectrometry (MS/MS), using hydrophilic interaction liquid chromatography (HILIC) for the detection of polar compounds and charged surface hybrid liquid chromatography (CSH) in positive and negative mode for the detection of lipids and nonpolar compounds. Gas chromatography (GC) coupled to TOF was used with Agilent 6890 GC Pegasus III TOF MS, to identify molecules of primary metabolism (amino acids, hexoses, phosphates, nucleotides, lipids and organic salts).

Biological samples were analyzed together, and the acquisition was performed in a randomized fashion. Sample preparation, data acquisition and analyses were performed following the routine and standard operational procedures of West Coast Metabolomics Center (University of California, Davis – USA), where the analysis were performed. Only known metabolites were included in this investigation.

### Clinical analysis

We assessed body composition, plasma biochemical and hormonal markers of glucose homeostasis (glucose, insulin, glucagon, glycated haemoglobin - HbA1c and C-peptide) and lipid profiles (low-density lipoprotein - LDL, very low-density lipoprotein - VLDL, high-density lipoprotein - HDL, cholesterol and triglycerides). Body composition was measured using air-displacement plethysmography (BOD POD^®^ COSMED, California, USA). Biochemical and hormonal markers of glucose homeostasis and lipid profiles, were analyzed at the Central Laboratory of the Hospital das Clinicas HCFMUSP, Faculdade de Medicina, Universidade de Sao Paulo (Sao Paulo, SP, Brazil) using enzymatic methods (glucose and lipid profiles), liquid chromatography (HbA1c), and electrochemiluminescence (insulin and C-peptide).

### Statistical analysis

Paired t and Wilcoxon tests were used to evaluate changes in body composition, biochemical and hormonal markers of glucose homeostasis and lipid profiles. Two-way ANOVA was used to investigate whether cholecystectomy induced significant changes in BAs via comparison of patients divided into two independent variables: time (before vs. after RYGB) and group (patients with cholecystectomy *vs*. patients without cholecystectomy). Therefore, we examinated the significance and impact of these two factors and their interaction. Comparisons were performed using R software. To provide an overview of general metabolic alterations induced by RYGB and the effect of cholecystectomy in this outcome, we used nonsupervised multivariate analysis (principal component analysis, PCA) and chemical similarity ontology mapping (ChemRICH)^12^ to the untargeted metabolomic data. By comparing pre and postoperative timepoints, PCA shows the magnitude of the variance and reveals the percentage of change. ChemRICH uses the Kolmogorov–Smirnov test and yields study-specific sets of all identified metabolites, generating clusters with chemical similarity and ontology mapping. A significance level of p ≤ 0.05 was used. Relative alterations were assessed as fold-changes to determine timepoint-relative alterations. Patients with and without cholecystectomy were analyzed together (general group) and as independent groups.

## Results

### Descriptive data of the patient population

Twenty-eight patients were included. The average age was similar between patients with (n = 7; 45 ± 7) and without (n = 21; 48 ± 7) cholecystectomy. Gallbladder removal was performed prior to (n = 3) or during RYGB (n = 4). The racial/ethnic composition included white, mixed race - brown (“pardo” Brazilians) and black, which totaled 42.8%, 42.8% and 14.2%, respectively, of patients with cholecystectomy and 76.1%, 19% and 4% in patients without cholecystectomy, respectively. Additional descriptive data of these subpopulations are shown in the preoperative columns of Table 1.

**Table 1:** Body composition and clinical parameters measured preoperatively and at 3 months postoperatively.

Variables	With cholecystectomy (n = 7)			Without cholecystectomy (n = 21)		
	Preoperative	Postoperative	p value	Preoperative	Postoperative	p value
Weight (kg)	117.28 ± 13.10	112.3 ± 10.89	<0.050	111.81 ± 16.20	108.17 ± 14.50	<0.050
BMI (kg/m^2^)	47.00 ± 4.60	45.80 ± 3.90	<0.050	44.40 ± 6.00	43.00 ± 5.30	<0.050
% Body Fat	50.30 ± 9.70	48.77 ± 4.70	**0.937**	51.83 ± 5.50	43.51 ± 6.30	**<0.050**
% Lean mass	49.60 ± 9.70	51.22 ± 4.70	**0.219**	48.17 ± 5.50	56.49 ± 6.30	**<0.050**
Body fat (kg)	59.10 ± 15.30	46.47 ± 8.00	**0.469**	57.96 ± 13.16	39.44 ± 9.90	**<0.050**
Lean mass (kg)	56.90 ± 7.80	48.53 ± 5.90	**0.297**	52.93 ± 6.80	50.16 ± 5.90	**<0.050**
Glucose (mg/dL)	195.40 ± 89.20	102 ± 27.70	<0.050	223.76 ± 67.80	102.68 ± 18.90	<0.050
Insulin (µU/mL)	18.10 (13.50–20,90)	7.8 (6.30–16.70)	<0.050	16.30 (13.45–25.40)	7.9 (6.90–12.70)	<0.050
%HbA1c	8.30 ± 1.90	5.9 ± 0.50	<0.050	9.08 ± 1.50	6.01 ± 0.40	<0.050
C peptide (ng/mL)	3.60 ± 0.90	2.64 ± 0.57	**0.109**	4.21 ± 1.40	3.01 ± 0.97	**<0.050**
Cholesterol total (mg/dL)	178.10 ± 42.10	151.28 ± 27.00	**0.297**	200.71 ± 32.40	166.84 ± 56.50	**<0.050**
HDL (mg/dL)	40.20 ± 11.08	44.14 ± 11.60	**0.202**	44.81 ± 9.35	42.10 ± 8.90	**<0.050**
LDL (mg/dL)	108.80 ± 36.50	87.85 ± 22.10	**0.352**	121.57 ± 33.62	101.2 ± 43.65	**<0.050**
VLDL (mg/dL)	29.00 ± 9.90	19.28 ± 3.30	0.156	29.58 ± 9.67	23.52 ± 10.54	0.078
Triglycerides (mg/dL)	145.10 ± 49.00	96.42 ± 16.80	**0.156**	175.5 ± 103	117.94 ± 52.70	**<0.050**

Data are expressed by mean ± standard deviation, except the data on insulin levels, which are expressed as median (interquartile ranges). Bold p values highlight the clinical variables that responded to RYGB at different significant levels in patients with or without cholecystectomy. BMI, body mass index; HbA1c%, glycated hemoglobin; HDL, high density lipoproteins; LDL, low density lipoproteins; VLDL, very low-density lipoproteins.

### Influence of cholecystectomy on plasma and fecal BAs response to RYGB

When considering patient subgroups according to the presence of cholecystectomy, we observed a distinct pattern of BA alterations after RYGB in plasma and fecal samples (Tables 2 and 3). Plasma samples of patients with cholecystectomy exhibited 3 increased BAs (GCDCA, TCDCA and TLCA) after RYGB (vs. preoperative) (p < 0.05). Patients with cholecystectomy exhibited 8 decreased fecal BAs (CA, GCDCA, GUDCA, GCA, LCA, TCA, TDCA and TLCA), and patients without cholecystectomy exhibited a reduction in only 4 BAs (CA, GCDCA, GUDCA and TUDCA). Comparisons of BAs profiles between these subgroups revealed that patients with cholecystectomy exhibited higher fecal and plasma GDCA and GLCA levels, lower fecal DCA and TDCA levels, and higher plasma TDCA, GCA and LCA levels than patients without cholecystectomy (p < 0.05).

**Table 2:** Comparison of fecal bile acid concentrations between obese female patients with and without cholecystectomy before and 3 months after Roux en-Y gastric bypass.

BA	Without cholecystectomy (n = 12)		With cholecystectomy (n = 7)		Anova p-value		
	Preoperative	Postoperative	Preoperative	Postoperative	Group	Time	Inter.
CA	260.00 (14.56–1690.00)	143.40 (12.46–884.00)	24.60 (4.20–33.60)	1.97 (0.98–4.70)	**<0.001**	**0.002**	0.270
CDCA	280.00 (18.18–872.00)	142.00 (48.20–718.00)	27.20 (3.04–100.00)	1.19 (0.84–2.90)	**<0.001**	0.120	0.251
DCA	852.00 (594.00–1884.00)	878.00 (418.00–2260.00)	1724.00 (1308.00–4700.00)	632.00 (384.00–1160.00)	0.924	**0.014**	**<0.001**
GCA	0.97 (0.37–10.74)	0.43 (0.16–1.20)	0.52 (0.38–1.10)	0.16 (0.09–0.30)	0.083	**0.002**	0.626
GCDCA	1.43 (1.00–7.78)	0.41 (0.23–1.99)	0.52 (0.50–1.09)	0.38 (0.21–0.72)	**0.040**	**<0.001**	0.218
GDCA	0.91 (0.01–5.62)	0.73 (0.41–3.88)	2.32 (1.70–4.82)	0.75 (0.61–1.21)	0.712	0.070	**0.034**
GLCA	0.21 (0.02–0.30)	0.12 (0.05–0.28)	0.36 (0.28–0.66)	0.17 (0.07–0.33)	0.233	**0.006**	**0.042**
GUDCA	0.19 (0.11–1.09)	0.09 (0.06–0.11)	0.16 (0.10–0.25)	0.07 (0.06–0.08)	**0.007**	**<0.001**	0.924
LCA	442.00 (7.20–930.00)	376.00 (87.80–438.00)	892.00 (480.00–2340.00)	432.00 (244.00–646.00)	0.120	**0.014**	0.286
TCA	0.77 (0.15–5.90)	0.07 (0.01–0.24)	0.19 (0.06–1.09)	0.07 (0.01–0.22)	0.388	**0.006**	0.523
TCDCA	1.62 (0.24–2.32)	0.03 (0.01–0.18)	0.13 (0.03–0.31)	0.01 (0.01–0.08)	**0.005**	**<0.001**	**0.016**
TDCA	0.23 (0.01–1.64)	0.10 (0.01–0.61)	0.84 (0.13–2.62)	0.04 (0.01–0.07)	0.966	**0.019**	0.216
TLCA	0.16 (0.01-0.30)	0.06 (0.01–0.12)	0.24 (0.09–0.46)	0.06 (0.01–0.09)	0.750	**0.003**	0.362
TUDCA	0.16 (0.10–1.12)	0.03 (0.01–0.04)	0.07 (0.04–0.11)	0.04 (0.04–0.05)	0.797	**<0.001**	0.211

Bile acids (BAs) are expressed in nM concentrations and their median interquartile ranges. Data were analyzed by two-way ANOVA. Significant differences (p ≤ 0.05) are bolded and reflect distinct BA profiles between the groups at the preoperative period (Group factor); decreases of specific BA from the preoperative to the postoperative within both groups (Group factor plus Time factor) or only one of the groups (Time factor); and distinct BA profiles between the groups at the postoperative period (Interaction [Inter.] factor). When significance occurs in Interaction factor, significances in Group and/or Time factors are statistically disregarded. CA, cholic acid; CDCA, chenodeoxycholic acid; DCA, deoxycholic acid; GCA, glycocholic acid; GCDCA, glycochenodeoxycholic acid; GDCA, glycodeoxycholic acid; GLCA, glycolithocholic acid; GUDCA, glycoursodeoxycholic acid; LCA, lithocholic acid; TCA, taurocholic acid; TCDCA, taurochenodeoxycholic acid; TDCA taurodeoxycholic acid; TLCA, taurolithocholic acid; and TUDCA, tauroursodeoxycholic acid.

**Table 3:** Comparison of plasma bile acid concentrations between obese female patients with and without cholecystectomy before and 3 months after Roux en-Y gastric bypass.

BA	Without cholecystectomy (n = 21)		With cholecystectomy (n = 7)		Anova p-value*		
	Preoperative	Postoperative	Preoperative	Postoperative	Group	Time	Inter.
CA	23.27 (10.58–80.20)	29.22 (17.05–140.97)	76.53 (28.41–175.61)	70.98 (34.25–82.77)	0.070	0.890	0.359
CDCA	91.46 (27.08–237.74)	45.17 (24.28–209.35)	143.29 (98.12–276.44)	166.39 (86.91–268.09)	**0.014**	0.799	0.942
DCA	373.63 (270.03–779.30)	334.66 (192.08–866.55)	225.62 (125.36–282.79)	327.32 (75.95–466.75)	0.156	0.792	0.182
GCA	103.82 (47.28–183.83)	130.10 (57.06–199.32)	61.79 (52.40–79.85)	324.31 (85.16–857.40)	0.640	**0.004**	**0.037**
GCDCA	285.71 (193.12–612.74)	498.47 (250.17–1238.31)	315.35 (205.93–453.63)	1460.71 (467.48–6308.14)	0.133	**<0.001**	0.140
GDCA	131.63 (66.55–196.91)	131.62 (79.26–454.16)	33.58 (13.81–144.73)	290.1 (143.98–385.08)	0.497	**0.001**	**0.009**
GLCA	4.74 (0.00–22.04)	4.64 (0.00–24.00)	0.00 (0.00–17.21)	27.06 (7.30–44.58)	0.339	**0.038**	**0.037**
GUDCA	19.31 (7.48–43.78)	22.37 (5.57–38.90)	31.07 (19.17–145.48)	55.98 (13.97–114.83)	**0.038**	0.828	0.935
LCA	20.42 (15.84–39.04)	11.06 (6.16–17.00)	14.25 (7.88–16.10)	18.15 (14.48–22.75)	0.877	0.548	**<0.001**
TCA	7.96 (0.00–19.20)	7.92 (0.00–18.51)	3.58 (1.33–14.00)	25.16 (1.43–46.48)	0.522	0.155	0.199
TCDCA	19.57 (14.26–34.57)	45.57 (18.25–112.28)	18.75 (7.10–69.63)	173.46 (21.66–309.58)	0.630	**0.004**	0.509
TDCA	10.22 (4.78–20.61)	13.04 (6.95–28.70)	2.47 (0.00–9.57)	16.02 (6.77–39.90)	0.227	**0.013**	**0.094**
TLCA	0.00 (0.00-0.00)	0.00 (0.00–2.18)	0.00 (0.00–1.11)	1.36 (0.00–2.02)	0.926	**0.020**	0.698
TUDCA	0.00 (0.00-0.00)	0.00 (0.00–2.43)	1.39 (0.00–1.47)	1.78 (0.00–3.70)	0.218	0.337	0.770

Bile acids (BAs) are expressed in nM concentrations and their median interquartile ranges. Data were analyzed by two-way ANOVA. Significant differences (p ≤ 0.05) are bolded and reflect distinct BA profiles between the groups at the preoperative period (Group factor); changes of specific BA from the preoperative to the postoperative within both groups (Group factor plus Time factor) or only one of the groups (Time factor); and distinct BA profiles between the groups at the postoperative period (Interaction [Inter.] factor). When significance occurs in Interaction factor, significances in Group and/or Time factors are statistically disregarded. CA, cholic acid; CDCA, chenodeoxycholic acid; DCA, deoxycholic acid; GCA, glycocholic acid; GCDCA, glycochenodeoxycholic acid; GDCA, glycodeoxycholic acid; GLCA, glycolithocholic acid; GUDCA, glycoursodeoxycholic acid; LCA, lithocholic acid; TCA, taurocholic acid; TCDCA, taurochenodeoxycholic acid; TDCA taurodeoxycholic acid; TLCA, taurolithocholic acid; and TUDCA, tauroursodeoxycholic acid −80.

### Influence of cholecystectomy on global metabolomics profile in response to RYGB

**Figure 1:**
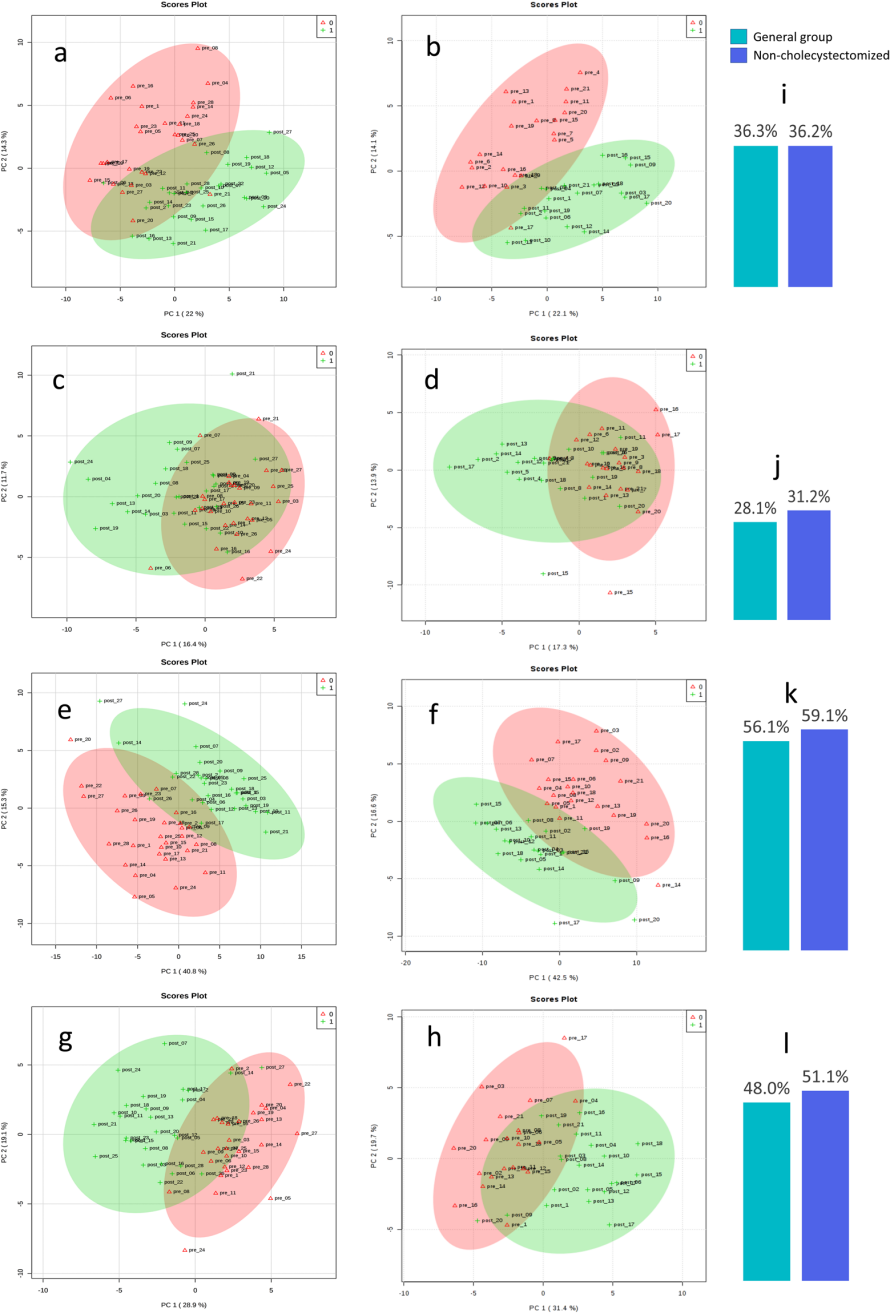
Principal component analysis of plasma untargeted metabolomics. The graphic displays phenotypic differences between samples collected before (red ellipses) and 3 months after RYGB (green ellipses). Patients are represented by red triangles (Δ) or green plus signals (+) and its position in the graphic is related to its individual metabolomic profile. Paired samples were named pre_ or post_ for preoperative and postoperative periods, followed by numbers from 1 to 28 to enable comparisons between the study points in time. Graphics were generated according the analytical platform: gas chromatography (GC), hydrophilic interaction liquid chromatography (HILIC), and charged surface hybrid liquid chromatography (CSH) in positive and negative mode. All included subjects are presented together as a general group (with and without cholecystectomy) and after exclusion of patients with cholecystectomy. Graphic disposition is presented for the general group analyzed using GC (**a**); patients without cholecystectomy analyzed using GC (**b**), general group analyzed using HILIC (**c**), patients without cholecystectomy analyzed using HILIC (**d**), general group analyzed using CSH in positive mode (**e**), patients without cholecystectomy analyzed by CSH in positive mode (**f**), general group analyzed using CSH in negative mode (**g**); patients without cholecystectomy analyzed using CSH in negative mode (**h**). Distance between preoperative and postoperative timepoints is proportional to the magnitude of the variance and reveals the percentage of change between pre and postoperative times. The percentage of metabolic changes are presented for GC (**i**), HILIC (**j**), and CSH in positive (**k**) and negative (**l**) modes.

Transformations of metabolomics profiles after RYGB were analyzed in patients with and without cholecystectomy in combination and as independent groups. When analyzed together (general group), the sum of PCA projections (PC1 and PC2) revealed that RYGB induced important alterations in several metabolite classes (Fig. 1). Overall, changes of 36.3%, 28.1%, 56.1% and 48% were detected using GC, HILIC, and CSH in positive and negative modes, respectively.

Analyses of only patients without cholecystectomy revealed minor intragroup variations compared to intergroup variations, and discrepancies between pre- and postoperative timepoints were increased (Fig. 1). Dataset alterations in this new organization totaled 36.2%, 31.2%, 59.1% and 51.1% in GC, HILIC, and CSH in positive and negative modes, respectively. Therefore, when compared to the combined group, the sum of these metabolic changes was a 9% increased.

When considering only patients with cholecystectomy, metabolic alterations after RYGB were even higher, and total changes of 48.3%, 41.7%, 54.2% and 47.2% in GC, HILIC, and CSH in positive and negative modes were observed, respectively. Therefore, when compared to the combined groups, the sum of metabolic changes induced by RYGB was increased 22.9%.

**Figure 2:**
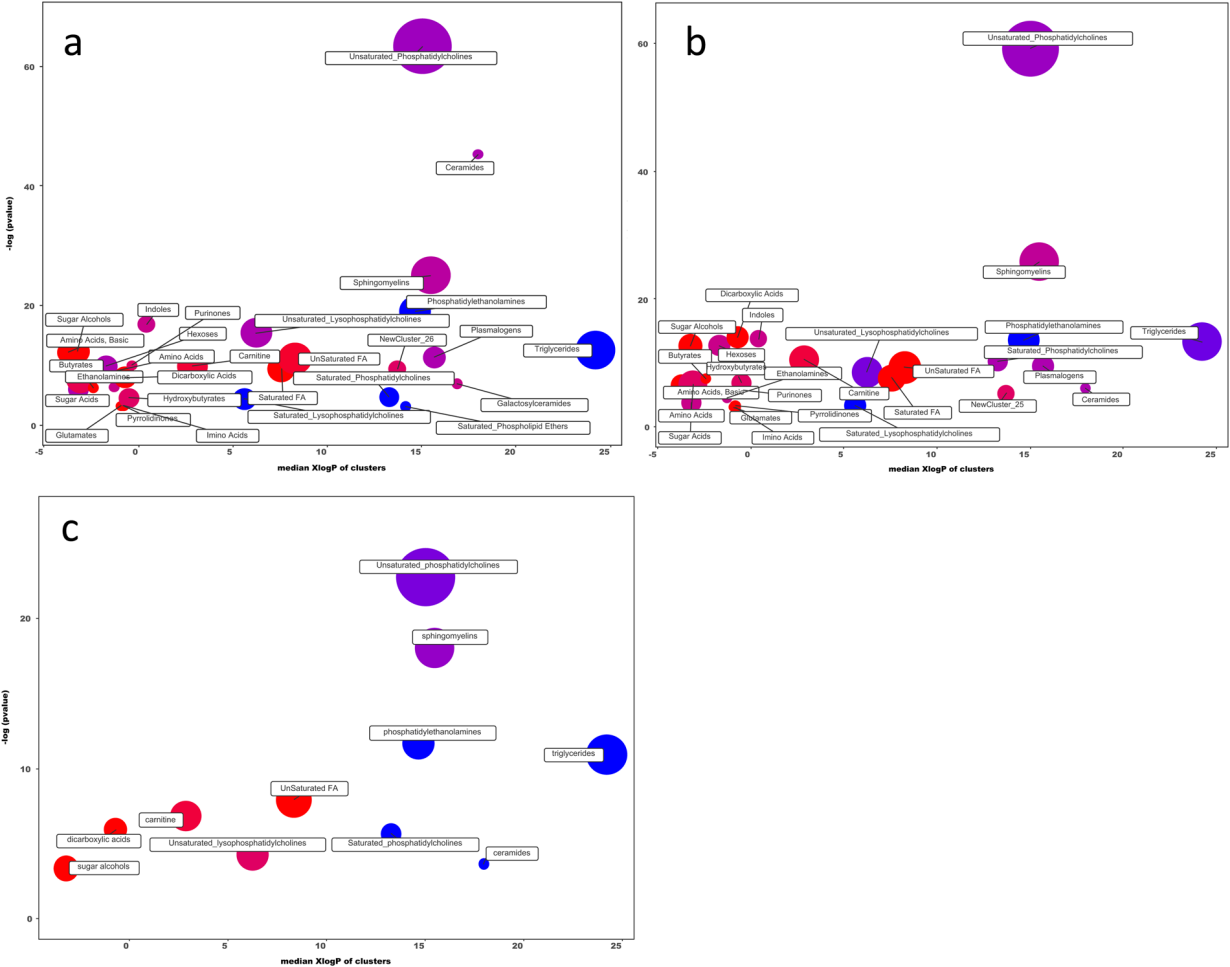
Chemical similarity ontology mapping of plasma untargeted metabolomics analysis. Comparisons of samples obtained before and 3 months after RYGB were made for patients with and without cholecystectomy, and analyzed together and as independent groups. Only significantly impacted metabolite clusters are displayed (p < 0,05). Plot y-axis shows the most significantly altered clusters on the top. Cluster colors give the proportion of increased or decreased compounds: increased in red, decreased in blue and both increase/decrease combined in purple. Patients with and without cholecystectomy are combined in a general group (**a**), and independent analysis are presented in patients without cholecystectomy (**b**) and patients with cholecystectomy (**c**). After removal of patients with cholecystectomy, metabolite alterations were different, particularly amino acids, carnitine, hexoses, saturated fatty acids, triglycerides and unsaturated phosphatidylcholines and lysophosphatidylcholines.

At the chemical level, cholecystectomy also impacted the RGYB-induced metabolomics alterations (Fig. 2). Chemical classification of the significantly altered metabolites (p < 0.05) showed a similar pattern between the general group and patients without cholecystectomy, but the key components, number of altered metabolites and the alterations (up or down) were different. Notably, the most altered metabolite classes did not reach statistical significance in patients with cholecystectomy. Therefore, despite the broader percentage of metabolic changes identified using PCA, the number of impacted metabolite classes was more restricted in this group. The entire list of the chemistry similarities is shown in Supplementary Table S1.

### Influence of cholecystectomy on the clinical response to RYGB

RYGB modified body composition, biochemical and hormonal markers of glucose homeostasis and lipid profile (Table 1). Comparisons of post- and preoperative timepoints revealed changes in body composition, particularly lean mass and body fat, and cholesterol, LDL and triglycerides were significantly improved only in patients without cholecystectomy. The VLDL lipoprotein was decreased in all patients, but the significance level was not reached in any of the studied populations for this variable. Fasting glucose, HbA1c and insulin were significantly decreased after RYGB in patients with and without cholecystectomy (p < 0.05). C-peptide was also decreased in both groups, but only patients without cholecystectomy reached the statistical significance (p < 0.05). Differences between these populations at the preoperative timepoint were found only for body weight, which was higher in patients with cholecystectomy. Six patients with cholecystectomy and 13 patients without cholecystectomy from our cohort achieved 1-year complete resolution of type 2 diabetes, according to de American Diabetes Association (ADA) criteria^13^.

## Discussion

Our study showed that cholecystectomy, which interferes with the BAs pool^14^, influenced the metabolic and clinical response to RYGB. At the metabolic level, this effect was marked by a distinct metabolomic profile and plasma and fecal BAs fluctuations between patients with and without cholecystectomy. Differences in BAs chemical structures can impact lipid and glucose homeostasis via FXR and TGR5 receptors and lead to several biological effects^15^. Therefore, cholecystectomy may influence changes in global systemic metabolism and affect clinical outcomes via modification of the BA response to RYGB.

The exclusion of patients with cholecystectomy or the division of patients according the presence of cholecystectomy emphasized metabolic differences between preoperative and postoperative timepoints, which improved the perception of the RYGB-induced metabolic changes. Therefore, the metabolic response of these subpopulations is different. Improvements in clinical markers were more frequent in patients without cholecystectomy than in patients with cholecystectomy, which suggests that cholecystectomy impairs the RYGB-related metabolic changes that contribute to better clinical outcomes. This impression is supported by the observation that postoperative changes in metabolite classes were poorest in patients with cholecystectomy than patients without cholecystectomy.

Notably, lipid subfractions of cholesterol, triglycerides and LDL were significantly improved only in patients without cholecystectomy, and this might be related to the BAs profile. BAs are a main product of cholesterol metabolism in the liver and exert profound effects on cholesterol and triacylglycerol, which regulates the metabolism of various lipoprotein species^16^. Cholecystectomy did not impair improvements of glycemic-related parameters, such as glucose, insulin and HbA1c, after RYGB, and patients achieved long-term diabetes remission. However, the alterations of BAs and global metabolomics data observed after RYGB suggest that the glycemic homeostasis achieved in patients with and without a gallbladder occurred via distinct metabolic pathways. This may include pathways that attenuate lipid-mediated insulin resistance (e.g. disturbed LDL) in patients without cholecystectomy because only this population exhibited significant relief in a marker of hyperinsulinemia (C-peptide)^17^.

Because subtle variations between individuals may result in large perturbations of metabolite concentrations and flux, and a minor stimulus may induce major metabolome changes^18^, the data presented here are particularly relevant. Independent changes in BAs concentrations may be induced by RYGB^19^ and cholecystectomy^14^. Our study demonstrated that the combination of these factors may result in the activation of specific metabolic pathways that are not activated when RYGB acts alone. Therefore, the present study investigated whether cholecystectomy produced parallel effects that acted as a confounding factor in assessments of RYGB metabolic consequences, particularly when studying T2D.

Clinical investigations of RYGB and metabolism should be designed to maintain consistency, reduce variation between subjects and optimize information retrieval. The application of cholecystectomy as an exclusion criterion will allow researchers to analyze more homogeneous populations and increase the biological interpretability of their data to draw more relevant and unbiased conclusions. The perception of metabolic shortcuts resulting from bariatric procedures may be improved via the separate investigation of patients with and without cholecystectomy. We hypothesized that the clinical benefits provided to patients with or without cholecystectomy after RYGB result from different mechanisms of action, triggered by different metabolic pathways.

Our study has some limitations that must be highlighted. The number of included patients with cholecystectomy was low, and this procedure was performed at heterogeneous timepoints, before or during the RYGB. However, comparisons were made with paired samples, and the discussed changes were related to the postoperative period. Our data showed a high phenotypic variance of complete biochemical profiles. Metabolomics data offer a comprehensive overview of metabolism that is well beyond the scope of standard clinical chemistry^20^. There are decades of precedence for the use of analyses of small numbers of metabolites to assess health and disease states, diagnose disease and impact clinical care (e.g., measuring glucose to monitor diabetes), but metabolomic changes reflect the main work of functioning cells, including the regulatory activities of the macromolecules in a complex feedback circuit^21^. Our populations groups were heterogenous in race. However, the reported race/ethnicity had a significant bias in studies with Brazilians because they have different perceptions of their race.For example, “pardos” race may be interpreted as white or black, depending on the perception of the interviewed subject. Therefore, it is difficult to determine whether race heterogeneity occurred between groups.

In conclusion, our study suggests that cholecystectomy affected the metabolic and clinical response to RYGB and became a source of bias in assessments of the effect of bariatric surgeries on metabolism and clinical outcomes of patients with obesity. We suggest the use of cholecystectomy as an exclusion criterion for patient selection or the stratification of patients according to the presence or absence of cholecystectomy. Because of the metabolic signaling properties of BAs, the possible interference of cholecystectomy on the metabolic effects of bariatric surgeries should be studied, primarily on glycemic homeostasis. These studies will improve our understanding of whether the time of cholecystectomy procedure (before, during or after the bariatric surgery) and its magnitude (how long before and after the bariatric surgery) may be an additional bias to understand its effects.

### Statement of informed consent

Informed consent was obtained from all individual participants included in the study.

### Statement of human and animal rights

All procedures performed in studies involving human participants were in accordance with the ethical standards of the institutional and/or national research committee and with the 1964 Helsinki declaration and its later amendments or comparable ethical standards.

## Supplementary information

Supplementary Table S1.
