# Author Correction: Cholecystectomy - a potential selection bias in studies assessing the metabolic effects of bariatric surgeries

**DOI:** 10.1038/s41598-021-86221-2

**Published:** 2021-03-17

**Authors:** Natasha Mendonça Machado, Camila de Siqueira Cardinelli, Tong Shen, Marco Aurélio Santo, Raquel Susana Torrinhas, Dan Linetzky Waitzberg

**Affiliations:** 1 Department of Gastroenterology, Laboratory of Nutrition and Metabolic Surgery of the Digestive Tract (LIM 35), Faculdade de Medicina FMUSP, Universidade de Sao Paulo, Sao Paulo, SP Brazil; 2 West Coast Metabolomics Center, University of California, Davis, CA United States; 3 Digestive Surgery Department. Hospital das Clinicas HCFMUSP, Faculdade de Medicina, Universidade de Sao Paulo, Sao Paulo, SP Brazil

Correction to: *Scientific Reports* 10.1038/s41598-020-66688-1, published online 30 June 2020

This Article contains errors in the average and standard deviation calculations of postoperative weight patients for the effect of Roux-en-Y gastric bypass reported in Table 1.

The ‘Weight (kg)’ value for ‘Postoperative’ patients ‘With cholecystectomy (n = 7),’

“112.3 ± 10.89”

should read:

“95 ± 10.55”

Additionally, the ‘Weight (kg)’ value for ‘Postoperative’ patients ‘Without cholecystectomy (n = 21),’

“108.17 ± 14.50”

should read:

“90.7 ± 12.40”

The correct Table 1 appears below:

**Table 1:** A corrected version of Table 1. Data are expressed by mean ± standard deviation, except the data on insulin levels, which are expressed as median (interquartile ranges). Bold p values highlight the clinical variables that responded to RYGB at different significant levels in patients with or without cholecystectomy. BMI, body mass index; HbA1c%, glycated hemoglobin; HDL, high density lipoproteins; LDL, low density lipoproteins; VLDL, very low-density lipoproteins.

Variables	With cholecystectomy (n = 7)			Without cholecystectomy (n = 21)		
	Preoperative	Postoperative	p value	Preoperative	Postoperative	p value
Weight (kg)	117.28 ± 13.10	95 ± 10.55	< 0.050	111.81 ± 16.20	90.7 ± 12.40	< 0.050
BMI (kg/m^2^)	47.00 ± 4.60	45.80 ± 3.90	< 0.050	44.40 ± 6.00	43.00 ± 5.30	< 0.050
% Body Fat	50.30 ± 9.70	48.77 ± 4.70	**0.937**	51.83 ± 5.50	43.51 ± 6.30	** < 0.050**
% Lean mass	49.60 ± 9.70	51.22 ± 4.70	**0.219**	48.17 ± 5.50	56.49 ± 6.30	** < 0.050**
Body fat (kg)	59.10 ± 15.30	46.47 ± 8.00	**0.469**	57.96 ± 13.16	39.44 ± 9.90	** < 0.050**
Lean mass (kg)	56.90 ± 7.80	48.53 ± 5.90	**0.297**	52.93 ± 6.80	50.16 ± 5.90	** < 0.050**
Glucose (mg/dL)	195.40 ± 89.20	102 ± 27.70	< 0.050	223.76 ± 67.80	102.68 ± 18.90	< 0.050
Insulin (µU/mL)	18.10 (13.50–20.90)	7.8 (6.30–16.70)	< 0.050	16.30 (13.45–25.40)	7.9 (6.90–12.70)	< 0.050
%HbA1c	8.30 ± 1.90	5.9 ± 0.50	< 0.050	9.08 ± 1.50	6.01 ± 0.40	< 0.050
C peptide (ng/mL)	3.60 ± 0.90	2.64 ± 0.57	**0.109**	4.21 ± 1.40	3.01 ± 0.97	** < 0.050**
Cholesterol total (mg/dL)	178.10 ± 42.10	151.28 ± 27.00	**0.297**	200.71 ± 32.40	166.84 ± 56.50	** < 0.050**
HDL (mg/dL)	40.20 ± 11.08	44.14 ± 11.60	**0.202**	44.81 ± 9.35	42.10 ± 8.90	** < 0.050**
LDL (mg/dL)	108.80 ± 36.50	87.85 ± 22.10	**0.352**	121.57 ± 33.62	101.2 ± 43.65	** < 0.050**
VLDL (mg/dL)	29.00 ± 9.90	19.28 ± 3.30	0.156	29.58 ± 9.67	23.52 ± 10.54	0.078
Triglycerides (mg/dL)	145.10 ± 49.00	96.42 ± 16.80	**0.156**	175.5 ± 103	117.94 ± 52.70	** < 0.050**

