# Supplementary material for: Cholecystectomy - a potential selection bias in studies assessing the metabolic effects of bariatric surgeries
Source: Sci Rep. 2020 Jun 30;10:10683. doi: 10.1038/s41598-020-66688-1 (PMC7327072; doi:10.1038/s41598-020-66688-1)
Supplement: Supplementary file 1 — Supplementary Table S1. [file 41598_2020_66688_MOESM1_ESM.docx]

**Cholecystectomy - a potential selection bias in studies assessing metabolic effects of bariatric surgeries**

Natasha Mendonça Machado^1^, Camila de Siqueira Cardinelli^1^, Tong Shen^2^, Marco Aurélio Santo^3^, Raquel Susana Torrinhas^1^, Dan Linetzky Waitzberg^1,3^.

1. Department of Gastroenterology, Laboratory of Nutrition and Surgery Metabolic of the Digestive Tract (LIM 35), Faculdade de Medicina FMUSP, Universidade de Sao Paulo, Sao Paulo, SP, Brazil;
2. West Coast Metabolomics Center, University of California, Davis, CA, United States;
3. Digestive Surgery Department. Hospital das Clinicas HCFMUSP, Faculdade de Medicina, Universidade de Sao Paulo, Sao Paulo, SP, Brazil;

**Correspondence to**: Camila de Siqueira Cardinelli: camilacardinelli@yahoo.com.br

Av. Dr. Arnaldo, 455, 2° andar, sala 2208 – Cerqueira César, CEP: 01246-903, São Paulo – SP, Brazil. Phone / Fax: +55 11 3061-7459.

**Table S1. Results of the chemical similarity ontology mapping of plasma untargeted metabolomics analysis.**

| **Cluster name** | **Cluster size** | **p-values** | | | **Key compound** | | | **Altered metabolites** | | | **Increased** | | | | **Decreased** | | | |
| --- | --- | --- | --- | --- | --- | --- | --- | --- | --- | --- | --- | --- | --- | --- | --- | --- | --- | --- |
|  |  | General | No  Chol | Chol | General | No  Chol | Chol | General | No  Chol | Chol | General | No  Chol | | Chol | | General | No  Chol | Chol |
| Amino Acids | 10 | <0.05 | <0.05 | 0.11 | Serine | Serine | Glycine | 4 | 3 | 3 | 3 | 2 | 2 | | 1 | | 1 | 1 |
| Amino Acids, Aromatic | 4 | 1 | 1 | 1 | Tryptophan | Tryptophan | N-(Linolenoyl)tyrosine | 1 | 1 | 0 | 0 | 0 | 0 | | 1 | | 1 | 0 |
| Amino Acids, Basic | 6 | <0.05 | <0.05 | 1 | N-dimethylarginine | N-dimethylarginine | N-dimethylarginine | 2 | 2 | 0 | 2 | 2 | 0 | | 0 | | 0 | 0 |
| Amino Acids, BC | 3 | 1 | 1 | 1 | Valine | Valine | Isoleucine | 0 | 0 | 1 | 0 | 0 | 0 | | 0 | | 0 | 1 |
| Amino Acids, Sulfur | 3 | 1 | 1 | 1 | Cystine | Cystine | Methionine | 0 | 0 | 0 | 0 | 0 | 0 | | 0 | | 0 | 0 |
| Butyrates | 3 | <0.05 | <0.05 | 1 | Threonic acid | Threonic acid | Isothreonic acid | 2 | 2 | 0 | 2 | 2 | 0 | | 0 | | 0 | 0 |
| Carnitine | 11 | <0.05 | <0.05 | <0.05 | AC (C18:1) | AC (C2:0) | AC (C18:1) | 7 | 6 | 6 | 6 | 5 | 5 | | 1 | | 1 | 1 |
| Ceramides | 3 | <0.05 | <0.05 | <0.05 | Cer (d18:1/23:0) | Cer (d18:1/23:0) | Cer (d18:1/23:0) | 3 | 3 | 2 | 1 | 1 | 0 | | 2 | | 2 | 2 |
| Dicarboxylic Acids | 6 | <0.05 | <0.05 | <0.05 | Aminomalonate | Fumaric acid | Aminomalonate | 5 | 5 | 2 | 5 | 5 | 2 | | 0 | | 0 | 0 |
| Diglycerides | 5 | 0.16 | 1 | 0.1 | Stearoyl LG | Stearoyl LG | DG (34:2) | 2 | 1 | 2 | 0 | 0 | 0 | | 2 | | 1 | 2 |
| Disaccharides | 4 | 1 | 1 | 1 | Cellobiose | Lactulose | Sucrose | 0 | 0 | 1 | 0 | 0 | 0 | | 0 | | 0 | 1 |
| Ethanolamines | 3 | <0.05 | <0.05 | 1 | Choline | Choline | Choline | 2 | 2 | 1 | 1 | 1 | 0 | | 1 | | 1 | 1 |
| Galactosylceramides | 3 | <0.05 | 1 | 1 | GlcCer (d42:2) | GlcCer (d42:2) | GlcCer (d42:2) | 2 | 1 | 0 | 1 | 1 | 0 | | 1 | | 0 | 0 |
| Glutamates | 3 | <0.05 | <0.05 | 1 | N-acetylglutamate | N-acetylglutamate | ABA | 2 | 2 | 0 | 2 | 2 | 0 | | 0 | | 0 | 0 |
| Hexoses | 6 | <0.05 | <0.05 | 0.33 | Glucose | Glucose | Fucose | 3 | 4 | 2 | 1 | 2 | 1 | | 2 | | 2 | 1 |
| Hydroxybutyrates | 5 | <0.05 | <0.05 | 1 | 2-deoxytetronic acid | 2-deoxytetronic acid | HBA | 3 | 3 | 1 | 2 | 2 | 0 | | 1 | | 1 | 1 |
| Imino Acids | 3 | <0.05 | <0.05 | 1 | HD | HD | Trans-4-hydroxyproline | 2 | 2 | 0 | 2 | 2 | 0 | | 0 | | 0 | 0 |
| Indoles | 4 | <0.05 | <0.05 | 1 | Indole-3-lactic acid | Indole-3-lactic acid | Indole-3-lactic acid | 4 | 4 | 1 | 2 | 2 | 0 | | 2 | | 2 | 1 |
| Keto Acids | 3 | 1 | 1 | 1 | 2-ketoisovaleric acid | 2-ketoisovaleric acid | 2-ketoiso-caproic acid | 0 | 0 | 0 | 0 | 0 | 0 | | 0 | | 0 | 0 |
| PE | 12 | <0.05 | <0.05 | <0.05 | PE (p-36:2) | PE (38:2) | PE (p-34:2) | 10 | 7 | 7 | 0 | 0 | 0 | | 10 | | 7 | 7 |
| Piperidines | 4 | 1 | 1 | 1 | Pipecolic acid | Pipecolic acid | Piperidone | 1 | 0 | 0 | 1 | 0 | 0 | | 0 | | 0 | 0 |
| Plasmalogens | 6 | <0.05 | <0.05 | 1 | PE (p-36:5) | PC (p-32:0) | PE (p-36:5) | 3 | 3 | 1 | 1 | 1 | 0 | | 2 | | 2 | 1 |
| Purinones | 3 | <0.05 | <0.05 | 1 | Theophylline | Uric acid | Hypoxanthine | 3 | 3 | 0 | 2 | 2 | 0 | | 1 | | 1 | 0 |
| Pyrrolidinones | 3 | <0.05 | <0.05 | 1 | Oxoproline | Pyroglutamic acid | Oxoproline | 2 | 2 | 1 | 2 | 2 | 1 | | 0 | | 0 | 0 |
| Saturated FA | 9 | <0.05 | <0.05 | 1 | Palmitic acid | Pelargonic acid | Myristic acid | 3 | 2 | 0 | 3 | 2 | 0 | | 0 | | 0 | 0 |
| Saturated LPC | 6 | <0.05 | <0.05 | 1 | LPC (14:0) | LPC (14:0) | LPC 140 | 2 | 2 | 1 | 0 | 0 | 0 | | 2 | | 2 | 1 |
| Saturated_PC | 5 | <0.05 | <0.05 | <0.05 | PC (30:0) | PC (30:0) | PC 280 | 3 | 3 | 4 | 0 | 1 | 0 | | 3 | | 2 | 4 |
| Saturated PLE | 3 | <0.05 | 1 | 1 | AGP | AGP | PC o-320 | 2 | 1 | 1 | 0 | 0 | 0 | | 2 | | 1 | 1 |
| SM | 20 | <0.05 | <0.05 | <0.05 | SM (d41:2) | SM (d41:2) | SM (41:2) | 15 | 16 | 13 | 6 | 7 | 3 | | 9 | | 9 | 10 |
| Sugar Acids | 5 | <0.05 | <0.05 | 1 | Glyceric acid | Glyceric acid | Glyceric acid | 3 | 2 | 1 | 1 | 1 | 1 | | 2 | | 1 | 0 |
| Sugar Alcohols | 7 | <0.05 | <0.05 | <0.05 | Myo-inositol | Myo-inositol | Lyxitol | 5 | 6 | 2 | 5 | 6 | 2 | | 0 | | 0 | 0 |
| Triglycerides | 21 | <0.05 | <0.05 | <0.05 | TG (46:0) | TG (46:0) | TG (44:1) | 12 | 9 | 10 | 0 | 1 | 0 | | 12 | | 8 | 10 |
| UnSaturated FA | 14 | <0.05 | <0.05 | <0.05 | Docosahexaenoic acid | Docosahexaenoic acid | Oleic acid | 9 | 5 | 4 | 8 | 5 | 4 | | 1 | | 0 | 0 |
| Unsaturated LPC | 12 | <0.05 | <0.05 | <0.05 | LPC (20:5) | LPC (20:5) | LPC (18:1) | 9 | 7 | 3 | 3 | 1 | 2 | | 6 | | 6 | 1 |
| Unsaturated PC | 49 | <0.05 | <0.05 | <0.05 | PC (32:1) | PC (38:5) | PC (30:1) | 41 | 39 | 21 | 11 | 10 | 3 | | 30 | | 29 | 18 |

Legend: Comparisons of samples obtained before and 3 months after RYGB for cholecystectomyzed and non-cholecystectomyzed patients, analyzed combined and as independent groups. Abreviations: ABA, 4-acetamido butyric acid; AC, acylcarnitine; AGP, acetyl-glycero-phosphocholine; BC, branched chain; Cer, ceramides; Chol, cholecystectomyzed patients; DG, diglycerides; FA, fatty acids; General, cholecystectomyzed and non-cholecystectomyzed patients combined; HBA 2-hydroxybutanoic acid; HD, Hydroxyproline dipeptide; LPC lysophosphatidylcholines; NoChol, non-cholecystectomyzed patients; PC, phosphatidylcholines; PE, phosphatidylethanolamine; PLE, phospholipid ethers; SM, sphingomyelins; Steatoyl LG, stearoyl-2-linoleoyl-sn-glycerol; TG, triglycerides.
